# Abnormal cortical thickness and structural covariance networks in systemic lupus erythematosus patients without major neuropsychiatric manifestations

**DOI:** 10.1186/s13075-022-02954-z

**Published:** 2022-11-28

**Authors:** Shu Li, Ru Bai, Yifan Yang, Ruotong Zhao, Bibhuti Upreti, Xiangyu Wang, Shuang Liu, Yuqi Cheng, Jian Xu

**Affiliations:** 1 Department of Rheumatology and Immunology, First Affiliated Hospital of Kunming Medical University, Kunming, China; 2 Department of Psychiatry, First Affiliated Hospital of Kunming Medical University, Kunming, China

**Keywords:** Systemic lupus erythematosus, Brain, Cortical thickness, Structural covariance networks, Graph theory, Clinical characteristics

## Abstract

**Background:**

Non-neuropsychiatric systemic lupus erythematosus (non-NPSLE) has been confirmed to have subtle changes in brain structure before the appearance of obvious neuropsychiatric symptoms. Previous literature mainly focuses on brain structure loss in non-NPSLE; however, the results are heterogeneous, and the impact of structural changes on the topological structure of patients’ brain networks remains to be determined. In this study, we combined neuroimaging and network analysis methods to evaluate the changes in cortical thickness and its structural covariance networks (SCNs) in patients with non-NPSLE.

**Methods:**

We compare the cortical thickness of non-NPSLE patients (*N*=108) and healthy controls (HCs, *N*=88) using both surface-based morphometry (SBM) and regions of interest (ROI) methods, respectively. After that, we analyzed the correlation between the abnormal cortical thickness results found in the ROI method and a series of clinical features. Finally, we constructed the SCNs of two groups using the regional cortical thickness and analyzed the abnormal SCNs of non-NPSLE.

**Results:**

By SBM method, we found that cortical thickness of 34 clusters in the non-NPSLE group was thinner than that in the HC group. ROI method based on Destrieux atlas showed that cortical thickness of 57 regions in the non-NPSLE group was thinner than that in the HC group and related to the course of disease, autoantibodies, the cumulative amount of immunosuppressive agents, and cognitive psychological scale. In the SCN analysis, the cortical thickness SCNs of the non-NPSLE group did not follow the small-world attribute at a few densities, and the global clustering coefficient appeared to increase. The area under the curve analysis showed that there were significant differences between the two groups in clustering coefficient, degree, betweenness, and local efficiency. There are a total of seven hubs for non-NPSLE, and five hubs in HCs, the two groups do not share a common hub distribution.

**Conclusion:**

Extensive and obvious reduction in cortical thickness and abnormal topological organization of SCNs are observed in non-NPSLE patients. The observed abnormalities may not only be the realization of brain damage caused by the disease, but also the contribution of the compensatory changes within the nervous system.

## Background

Systemic lupus erythematosus is a multisystem connective tissue disease mediated by autoimmunity. When it involves the nervous system, neurological symptoms and mental disorders can occur, which is known as neuropsychiatric lupus erythematosus (NPSLE). Its symptoms range from obvious manifestations such as stroke, epilepsy, and myelopathy to subtle abnormalities such as anxiety and cognitive disorders [1]. NPSLE may be the result of interactions between immune pathological processes such as autoantibodies, intrathecal inflammatory mediators, cerebrovascular diseases, genetic factors, and blood-brain barrier destruction [2, 3]. It is considered to be the most serious form of organ involvement and a poor prognostic factor, and can significantly reduce the life expectancy of patients [4].

Many researchers, including our research team, define systemic lupus erythematosus without major neuropsychiatric symptoms such as SLE patients who have not been previously diagnosed with NPSLE, have no mental disorders such as anxiety or depression, and have not shown obvious neuropsychiatric symptoms and no obvious abnormalities in conventional magnetic resonance; also termed, non-neuropsychiatric systemic lupus erythematosus (non-NPSLE) [5]. At present, there is no single clinical, laboratory, neuropsychological, or imaging technique that can distinguish NPSLE from non-NPSLE patients with mild neuropsychiatric symptoms [6, 7]. Previous studies have found that 31% of non-NPSLE patients were confirmed to have moderate cognitive dysfunction even in the absence of obvious neuropsychiatric symptoms [8]. Several studies performed MRI scans and analyses of the brains of non-NPSLE patients and found structural and functional abnormalities [5, 9–11]. These results prove that non-NPSLE causes changes in the central nervous system before it develops into NPSLE. So, targeted research on non-NPSLE may be more helpful for understanding the pathological mechanism of SLE and reducing the burden on patients.

The cerebral cortex is composed of a large number of neuron cells and dendrites. Cortical thickness (CT) refers to the distance from the surface of the pia mater to the interface between white and gray matter. Cortical thickness varies from region to region, with normal cortical thickness ranging from 2 to 4mm [12]. Cortical thickness changes are frequently observed in certain pathological conditions. For example, Alzheimer’s disease shows cortical thinning, while Cortical dysplasia shows cortical thickening [13, 14]. Changes in cortical thickness are usually region-specific and vary between diseases and at different stages of the same disease [15]. Therefore, it can provide objective observation indicators for neuropsychiatric diseases, which is helpful for morphological identification between diseases and staging of diseases.

FreeSurfer is currently the most commonly used software package for analyzing brain cortical data and has been proven to have good test-retest reliability [16]. Currently, there are two main strategies for MRI-based cerebral cortex research. First is region-wise analysis, which includes analysis based on prior regions of interest (ROIs) or exploratory whole-brain analysis of regions defined by brain partition atlas, such as Desikan–Killiany (DK) atlas [17] and Destrieux (DS) atlas [18]. Another widely used strategy is to analyze cortical structural data from all vertices throughout the brain, termed surface-based morphometry (SBM), which is not restricted to prior structural regions. These two strategies have their advantages and disadvantages. Superiority of either in reflecting the underlying pathophysiology of the disease has not yet been determined. At present, several papers have described the use of SBM to study the cerebral cortex of SLE, which found abnormal cortical thickness in SLE patients [9, 19, 20]. However, use of ROIs to study the thickness of SLE cerebral cortex has not been reported. The correlation of brain structures across different regions is known as structural covariance, also called structural covariance networks (SCNs) [21]. Regions with high structural covariance may share synchronized developmental changes due to direct axonal connections or mutual influence, so strong regional intercortical thickness correlations are considered a measure of intercortical structural connectivity [21]. The difference in SCNs under different health conditions can be used to reflect the impact of specific environments on the brain created by experiences such as brain development, disease states, and social environment [22–24]. Studies based on SCNs have been reported for the research of obsessive-compulsive disorder [25], Alzheimer’s disease [26], Parkinson’s diseases [27], complete thoracic spinal cord injury [28] and other diseases. It also provides new methods for understanding the underlying pathophysiological mechanisms of brain developmental processes and diseases.

A branch of mathematics is known as graph theory. In graph theory, graphs are composed of many nodes and edges. Such diagrams usually describe specific relationships between certain objects. Currently, many studies are using graph theory to organize the brain as a network of nodes and edges and to explore brain networks by analyzing the properties of graph theory [29, 30].

The first purpose of this study was to compare the cortical thickness of 108 non-NPSLE patients and 88 healthy controls using ROIs and SBM method, respectively. As far as we know, this is the first study using these two analytical methods on SLE cerebral cortex, providing an opportunity to comprehensively describe the cortical changes of SLE and compare the similarities and differences between the results of the two methods. The second purpose of this study is to determine the relationship between abnormal cortical thickness in SLE and clinical indicators such as disease activity, mental scale, autoantibodies, and treatment status. Finally, we further applied the graph theory network to analyze the changes of the cortical thickness network in SLE patients. Using an exploratory approach, we measured and compared cortical thickness and its SCNs in a large sample size of non-NPSLEs and HC to comprehend the features of the cerebral cortex in SLE patients and their underlying physiological and pathological mechanisms.

## Materials and methods

### Participants

A total of 115 non-NPSLE patients (case group) were recruited from the outpatient and inpatient departments of the Rheumatology and Immunology Department of First Affiliated Hospital of Kunming Medical University.

The inclusion criteria for the case group: (1) According to the 1997 revised American College of Rheumatology (ACR) criteria for SLE, patients are classified as having the disease; (2) age range from 18 to 50 years; (3) Han ethnicity; (4) right-handedness.

The exclusion criteria for the case group were as follows: (1) patients with connective tissue disorders such as systemic sclerosis, primary or secondary Sjögren’s syndrome, or systemic sclerosis who meet the ACR classification criteria; (2) patients with epilepsy, severe active mental illness, stroke, traumatic brain injury, history of intracranial surgery, etc. that brain structures or functional imaging may be affected; (3) patients with a history of drug abuse and alcoholism; (4) women during pregnancy or lactation; (5) MRI contraindications (e.g., metal implants, claustrophobia); (6) patients with structural brain abnormalities on conventional T1- or T2-weighted MRI scan.

Three volunteers refused MRI, one volunteer showed lacunar infarction on MRI, and three had partial MRI results missing. Eventually, 108 non-NPSLE patients were enrolled.

The inclusion criteria for healthy controls (HCs) were as follows: (1) subjects in good health; (2) age- and gender-matched with the patient group; (3) Han ethnicity; (4) right-handedness.

The exclusion criteria for HCs were as follows: (1) history of mental illness or family history of psychosis; (2) subjects who have an organic encephalopathy or neurological disorder; (3) subjects with a history of mental illness or a family history of mental illness; (4) subjects with a history of drug abuse and alcoholism; (6) subjects who have contraindications to MRI; (7) subjects with structural brain abnormalities on conventional T1- or T2-weighted MRI scan.

Eighty-eight HCs were recruited.

The Ethics Committee of First Affiliated Hospital of Kunming Medical University has approved this study. Informed consent was obtained from the subjects and their legal guardians before the trial began.

#### Clinical characteristics of SLE patients

The age, gender, duration (time from SLE onset to completion of MRI examination), cumulative doses of glucocorticoids (GC), cyclophosphamide (CTX), and hydroxychloroquine (HCQ) in non-NPSLE patients were registered. Symptoms and signs were recorded in detail.

#### Immunology test

Autoantibodies such as anti-nuclear antibody (ANA), anti-Smith antibody (ASmA), anti-double-stranded DNA (anti-dsDNA) antibody, anti-ribosomal P0 antibody (ARPA), lupus anticoagulant material (LAC) and anti-cardiolipin antibody (ACL) were examined using indirect immunofluorescence (IIFA) and immunoblotting. Then, 24 h urine protein was recorded.

#### Disease activity assessment

SLEDAI-2k scores quantified disease activity as follows: a score between 0 and 4 was defined as inactive state, between 5 and 9 represented light activity, the score of 10 and 14 was classified as moderate activity, and more than 15 was classified as severe activity [31, 32].

#### Psychological assessment scale

Each subject’s cognitive function, anxiety and depression were assessed by a psychiatrist on the day of the MRI examination using Mini-Mental State Examination (MMSE), Hamilton Depression Scale (HAMD) and Hamilton Anxiety Scale (HAMA).

### Image processing

An experienced neuroradiologist acquired MRI images of all subjects using a 1.5-T MRI scanner with head coils. Initially, T1WI and T2WI were performed to exclude obvious structural abnormalities. No subjects were excluded due to obvious structural abnormalities. 3D-MRI uses 3D-T1 weighted fast phase disturbance gradient echo sequence (3D-T1-fspgr sequence), and the parameters were as follows: repetition time (TR) = 10.5 ms, echo time (TE) = 2 ms, inversion time = 350 ms, layer thickness = 1.8 mm and no layer interval, scanning matrix = 256×256, flip angle (FA) = 15°, spatial resolution = 0.94 mm×0.94 mm×0.9 mm, layer number = 172, scans cover the entire brain.

Using FreeSurfer package (version 5.3.0, https://surfer.nmr.mgh.harvard.edu/fswiki/DownloadAndInstall#Download) to process structural images. Previous publications have described the details of the image processing procedure [33]. In brief, processing comprises raw data import, data format conversion, head motion correction, automatic Talairach transformation, non-uniform field correction, skull removal, brain tissue segmentation, and automatic reconstruction of pia mater and gray and white matter surfaces. The next step was to resample the data and smooth it onto FreeSurfer average subject templates. For group-level whole-brain analysis, a 10 mm surface-based full-width-at-half-maximum (FWHM) Gaussian kernel map was filtered before statistical analysis. Participants’ final maps were averaged using a non-rigid high-dimensional spherical approach to align cortical folding patterns [34]. FreeSurfer Qoala-T tools were utilized [35] to evaluate the results of cortical segmentation for the influence of head motion, the reconstruction of key regions, and the mis-segmentation of non-brain tissue. All segmentation results meet the verification requirements.

#### Cortical thickness calculation

For SBM method, according to the principle of SBM, the FreeSurfer uses the grid-based surface analysis method proposed by Fishl et al. [36]. This technique method defines cortical thickness as the shortest distance from the white matter-gray matter interface to the gray matter-soft brain matter interface. The average of the shortest distance from the outer to the inner surface and vice versa at a predefined site on the vertex of the outer surface called the origin, refers to the thickness of the cortex at the vertex. For ROI method, cortical thickness was computed for 74 bilateral Destrieux [18] atlas regions by extracting the average value of the cortical thickness of all vertices in each segmented region using the script provided by the FreeSurfer [36].

### Construction of structural covariance networks

SCN analysis is done using Graph Analysis Toolbox [37, 38]. By treating the 148 brain regions of the Destrieux atlas as ROIs and defining them as nodes, Pearson’s correlation between the cortical thickness of each pair of ROIs is defined as the edges of the SCNs, with gender and age as covariates, a 148 × 148 association matrix M of each group is derived, and each entry rij is defined as Pearson’s correction coefficient between the cortical thickness of the regions i and j. The comparison of graph measures requires a minimum graph density to ensure that the two graphs being compared are fully connected (non-fragmented). According to calculations, the minimum density of the matrix constructed was 0.38; above 0.38, each node in the networks of the non-NPSLE group and the HC group has at least one connection to another node in the respective network. Densities above 0.5 may not be biologically meaningful for structural networks. Therefore, this study sets the range from 0.38 (Dmin) to 0.5 (Dmax) with an interval of 0.01 as the density of the network [38, 39]. Matrix A was derived from each association matrix where aij was considered 1 if rij > 0.38 (Dmin) and zero otherwise. Zero was also set for the diagonal elements of the association matrix. For more information, see Hosseini et al. [38, 40].

### Network properties

Firstly, we evaluated the differences in global network properties within and between groups, including characteristic path length, clustering coefficient, global efficiency, transitivity and modularity, and small-world index. Secondly, the local efficiency, betweenness centrality, and nodal degree are calculated as regional network parameters to describe the topological characteristics of SCNs. In short, the clustering coefficient is the ratio of the actual number of edges between nodes in the neighborhood to the number of edges that may exist between them and describes the degree of interconnection between nodes. The characteristic path length is the average of the shortest paths between pairs of nodes; the longer the path length, the lower the information transmission efficiency. Global efficiency is the inverse of the harmonic mean of the shortest path length between nodes. The small-world index is defined as the ratio of normalized clustering coefficient to the normalized characteristic path length. Transitivity expresses the globality of the clustering coefficient, which represents the aggregation degree of a network as a whole. Modularity reflects the extent to which the network can be decomposed into sub networks (modules) with maximum intramodule connections and minimum intermodule connections. The local efficiency of a node is the global efficiency of the subgraph composed of its nearest neighbors. The nodal degree describes the number of edges that are associated with a node. Betweenness centrality is a measure of a node’s centrality in the network equal to the proportion of all shortest paths through a given node to the total number in the network [37, 41, 42]. The hub of the network is the node that plays a key role in the exchange of information in the network. In this study, hubs are defined as regions where the nodal betweenness centrality is at least 2 standard deviations greater than the average nodal betweenness of the network.

### Statistical analyses

Non-parametric K-S test used to test normality of the data and the data distributed normally were expressed as mean ± standard deviation ($\overline{\textrm{x}}\pm s$), while the data with skewed distribution were expressed as median (p25%, p75%). An independent two-sample *T*-test was used to compare the age difference between the two groups. Chi-square test (*χ*^2^) was used to compare the gender composition ratio between the case group and the control group. For each test statistic, a two-tailed probability value of < 0.05 was considered as significant. SMB analyses: standard general linear model (GLM) was used to detect significant differences in cortical thickness between the two groups (corrected for the effects of gender and age). Then, a Monte-Carlo simulation cluster analysis with 1000 permutations, cluster-wise threshold of *P* < 0.05, and cluster-forming threshold of *P* < 0.001 were adopted to correct for multiple comparisons. Results were displayed via Freeview. For ROI analyses, all data were analyzed by SPSS 23.0 software package (SPSS, Inc, Chicago, IL). The average cortical thickness of the 148 brain regions of each subject in the two groups according to the DS atlas was extracted separately and the difference was tested by using two independent samples *T*-test. The threshold for statistical significance was set at *P* < 0.00033784 (0.05/148) to correct for multiple comparisons (Bonferroni correction). Correlation analysis was conducted between the clinical data and the cortical thickness of brain regions with statistically significant differences in ROI analysis. Among them, SLEDAI score, MMSE, HAMD, HAMA course of disease, ANA titer, ACL, LAC, CTX, HCQ accumulation, and mean cortical thickness of abnormal brain region were analyzed by Spearman correlation. The Point biserial correlation is used to analyze the correlation between autoantibodies (negative or positive) and cortical thickness. The criterion for statistical significance was *P* < 0.05.

## Results

### Demographics

**Fig. 1:**
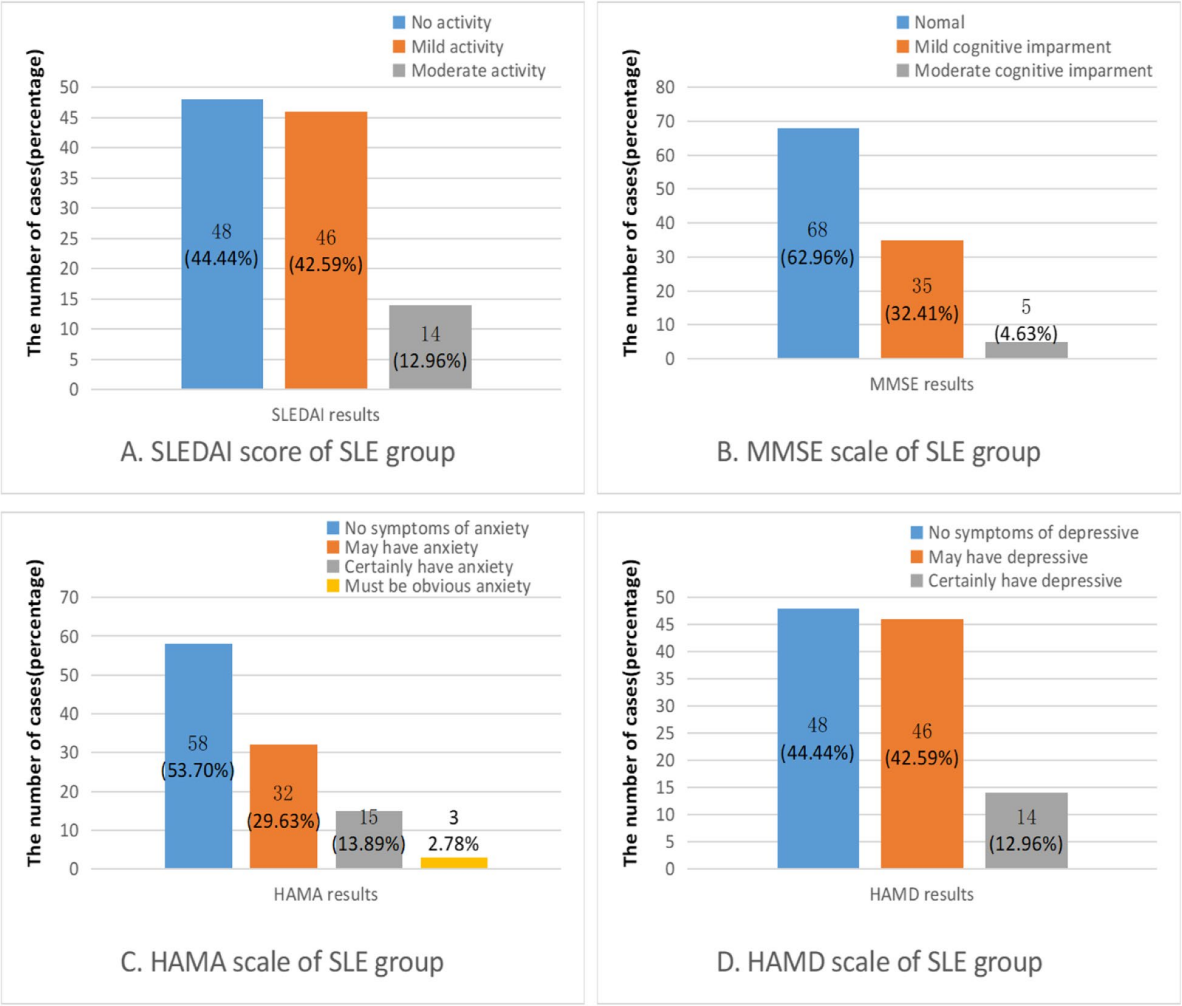
SLEDAI score (**A**), MMSE (**B**), HAMA (**C**), and HAMD (**D**) rating results

One hundred and eight non-NPSLE patients and 88 healthy controls (HCs) were included. The non-NPSLE groups and HC groups were matched for age (*t*=−1.506, *P* =0.134) and gender (*χ*^2^=3.256, *P* =0.071). Non-NPSLE patients received varying drug regimens, 84 patients had received glucocorticoids, 26 had received CTX, and 46 had received HCQ. LAC and ACL tests were performed in most patients (*n*=79). Table 1 shows the demographic information and some clinical characteristics of the two groups. SLEDAI scores of the patients in the case group, MMSE, HAMA, and HAMD rating results are shown in Fig. 1, antibodies to extractable nuclear antigens results are shown in Table 2.

**Table 1:** Results of demographic and clinical data of non-NPSLE group and HCs

	non-NPSLE (*n*=108)	HCs (*n*=88)	*t*/*χ*^2^-value	*P*-value
Gender (%)			3.256	0.071
Male	19(17.6)	25(28.4)		
Female	89(82.4)	63(71.6)		
Age (year)	29.49±6.78	31.06±7.76	−1.506	0.134
course of disease (month)	12.00(3.00,24.00)	/	/	/
Glucocorticoid accumulation(g)	5.59(0.77,11.22) (*n*=84)	/	/	/
CTX accumulation(g)	2.60(1.00,7.55) (*n*=26)	/	/	/
HCQ accumulation(g)	9.40(2.00,111.00) (*n*=46)	/	/	/
ACL	9.22(6.10,13.00) (*n*=79)	/	/	/
LAC	13.70(6.81,22.80) (*n*=79)	/	/	/
SLEDAI	9.00(5.25,14.00)	/	/	/
MMSE	28.00(25.00,29.00)	/	/	/
HAMA	5.00(3.25,10.00)	/	/	/
HAMD	7.00(3.25,13.75)	/	/	/

**Table 2:** Results of antibodies to extractable nuclear antigens in the non-NPSLE group (*n*=108)

Antibodies	Positive(%)	Negative(%)
Anti-dsDNA antibodies	74(68.5)	34(31.5)
Anti-Sm antibodies	50(46.3)	58(53.7)
Anti-U1RNP antibodies	33(30.6)	75(69.4)
Anti-SSA52KD antibodies	54(50.0)	54(50.0)
Anti-SSA60KD antibodies	66(61.1)	42(38.9)
Anti-SSB antibodies	38(35.2)	70(64.8)
Anti-Histones antibodies	60(55.6)	48(44.4)
Anti-P0 antibodies	55(50.9)	53(49.1)
Anti-Nucleosome antibodies	44(40.7)	64(59.3)
Anti-Centromere antibodies	3(2.8)	105(97.2)
Anti-DNP antibodies	13(12.0)	95(88.0)

### Between-group comparison of cortical thickness (SBM analyses)

**Fig. 2:**
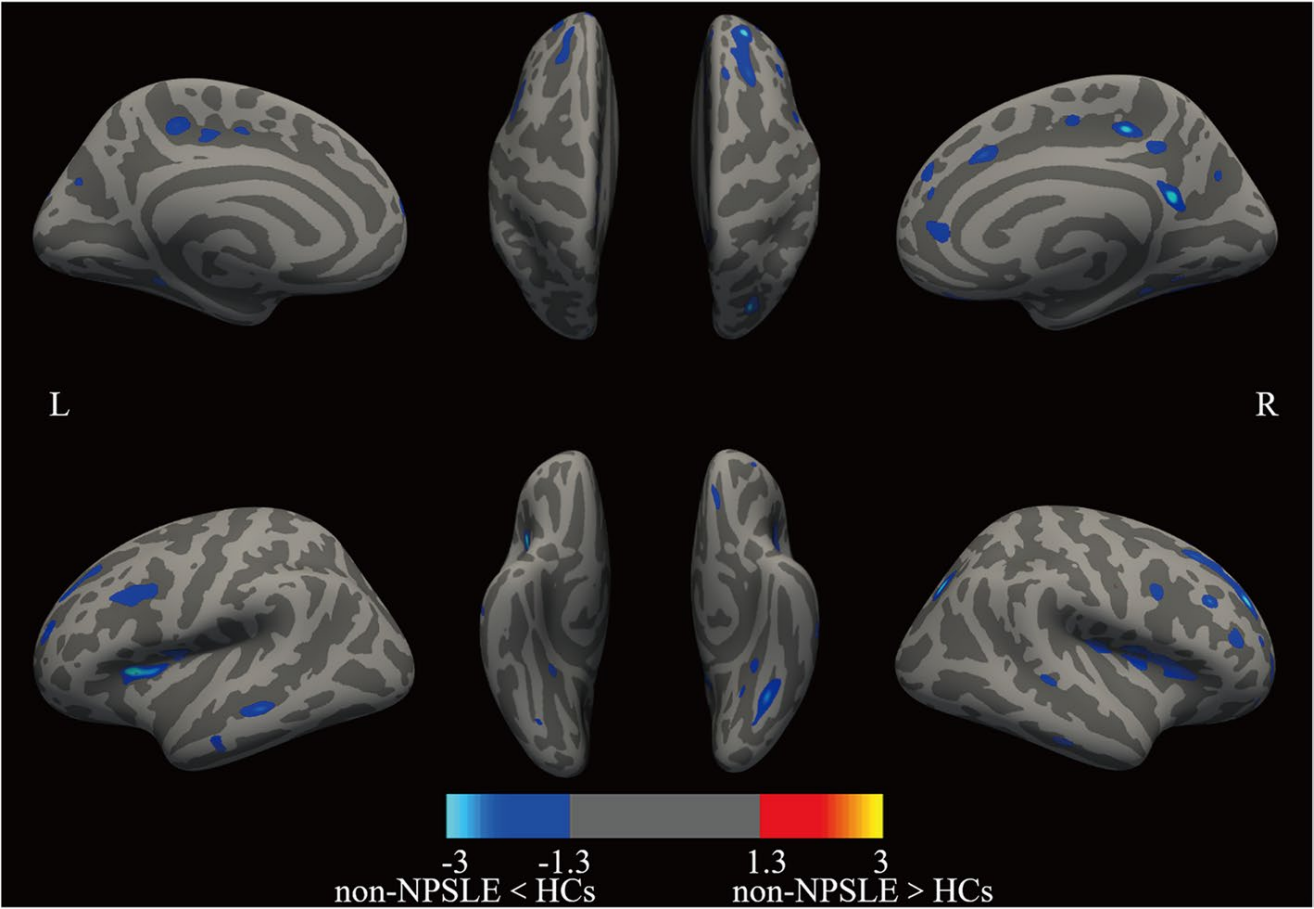
SBM comparison of non-NPSLE group to HCs after multiple comparison correction. Images showed clusters of decreased cortical thickness (blue cluster) in the non-NPSLE group compared with the HCs. None of the clusters showed increased cortical thickness in the non-NPSLE group compared with the HC group. Clusters are displayed in the range of *P* ≤0.05 to *P* ≤0.001 (colorbar represents −log (10) *P*-value, after Monte-Carlo correction for multiple comparisons). HCs, healthy controls; L, left cerebral hemisphere; R, right cerebral hemisphere

Compared with HCs, the non-NPSLE group had 18 clusters in the left hemisphere and 16 clusters in the right hemisphere, showing a significant reduction in cortical thickness. All clusters survived the Monte-Carlo correction for multiple comparisons. We did not find any clusters in the non-NPSLE group whose cortical thickness was significantly thicker than that of HCs. Projections of clusters with significant differences in cortical thickness were made on the brain surface template (Fig. 2, Table 3).

**Table 3:** Clusters changes in cortical thickness in non-NPSLE group compared with HCs (SBM analyses)

Contrast		Cortical regions	Size (mm^2^)	xyz-peak (MNI)	*P*-value
non-NPSLE<HCs	Left	S_circular_insula_sup	2941.56	−31.0/20.3/12.5	0.0001
		S_front_middle	2152.76	−27.6/45.3/14.1	0.0001
		S_cingul-Marginalis	1469.93	−18.7/−38.9/44.8	0.0001
		S_front_inf	1101.96	−40.3/26.1/16.8	0.0001
		S_temporal_sup	878.23	−55.3/−32.0/−11.2	0.0001
		G_oc-temp_lat-fusifor	853.72	−31.3/−68.7/−14.9	0.0001
		S_temporal_sup	794.76	−37.9/−55.4/18.9	0.0001
		S_parieto_occipital	737.21	−17.6/−75.1/23.7	0.0001
		S_front_sup	545.85	−21.7/17.6/53.8	0.0001
		G_rectus	526.56	−4.5/46.8/−19.8	0.0001
		G_front_sup	451.51	−7.7/29.9/45.9	0.0001
		S_orbital-H_Shaped	441.84	−19.5/28.3/−15.4	0.0001
		S_intrapariet_and_P_trans	436.74	−17.3/−70.7/40.5	0.0001
		G_pariet_inf-Supramar	348.93	−50.4/−46.0/44.8	0.0003
		G&S_paracentral	311.81	−6.1/−42.7/68.4	0.0006
		G_occipital_middle	306.69	−41.5/−83.2/4.2	0.0007
		S_oc-temp_med_and_Lingual	188.53	−30.4/−41.9/−8.6	0.0123
		S_intrapariet_and_P_trans	144.67	−27.0/−56.3/39.9	0.0397
	Right	G_front_middle	9634.23	26.1/42.8/31.6	0.0001
		G_oc-temp_lat-fusifor	2804.26	33.0/−57.6/−15.0	0.0001
		G_precuneus	1967.74	6.2/−54.8/16.6	0.0001
		G_pariet_inf-Angular	845.03	34.2/−61.6/44.7	0.0001
		S_orbital_med-olfact	630.71	10.2/35.3/−19.9	0.0001
		G&S_cingul-Mid-Ant	552.84	13.0/19.3/37.2	0.0001
		S_oc_sup_and_transversal	479.05	27.8/−65.3/26.3	0.0001
		S_parieto_occipital	392.62	18.5/−64.3/6.0	0.0001
		S_occipital_ant	279.71	43.4/−64.3/31.5	0.0005
		G_temp_sup-Plan_polar	245.21	53.6/0.1/−7.9	0.0025
		S_precentral-sup-part	240.50	22.6/−8.1/49.6	0.0031
		G_temp_sup-Plan_tempo	215.61	61.9/−22.8/5.8	0.0067
		G_precuneus	203.79	6.8/−50.7/60.3	0.0088
		S_oc_middle_and_Lunatus	180.95	42.2/−78.2/6.2	0.0160
		S_temporal_sup	167.49	49.0/−30.0/1.2	0.0221
		Pole_occipital	139.25	13.2/−101.2/8.2	0.0439
non-NPSLE>HCs	-	-	-	-	-

Based on Destrieux atlas with 148 brain regions. *HCs*, healthy controls; *MNI*, Montreal Neurological Institute

### Between-group comparison of cortical thickness (ROI analyses)

The ROIs was divided according to the DS atlas and the average cortical thickness of each region of the two groups was extracted for two independent samples *T*-test. The cortical thickness of 57 regions (27 in the left hemisphere and 30 in the right) in the non-NPSLE group was found to be decreased compared with that in the HC group (all *P* values < 0.00033784, Table 4). As for the results of SBM analysis, we found no increased cortical thickness in the non-NPSLE group compared with the HC group.

**Table 4:** Regions changes in cortical thickness in non-NPSLE group compared with HCs (ROI analyses)

Brain regions of Destrieux atlas	Cortical thickness(mm,$\overline{\textrm{x}}\pm s$)		*P*-value
	non-NPSLE (*n*=108)	HC(*n*=88)	
Left			
G&S_paracentral	2.43±0.14	2.52±0.14	2.44E−05
G&S_subcentral	2.50±0.14	2.58±0.16	1.51E−04
G&S_cingul-Mid-Post	2.53±0.14	2.62±0.14	7.91E−06
G_front_inf-Triangul	2.55±0.19	2.65±0.17	2.57E−04
G_front_sup	2.93±0.16	3.02±0.14	1.54E−05
G_occipital_middle	2.43±0.14	2.52±0.15	2.22E−05
G_occipital_sup	2.08±0.14	2.18±0.17	3.74E−05
G_pariet_inf-Supramar	2.57±0.12	2.64±0.14	2.50E−04
G_precuneus	2.41±0.22	2.50±0.24	3.15E−05
G_temporal_middle	2.84±0.19	2.94±0.16	1.70E−04
S_cingul-Marginalis	2.16±0.12	2.26±0.14	2.20E−07
S_circular_insula_ant	2.64±0.16	2.74±0.19	1.00E−04
S_circular_insula_sup	2.43±0.11	2.55±0.10	2.54E−12
S_front_inf	2.16±0.13	2.25±0.12	1.99E−07
S_front_middle	2.20±0.16	2.31±0.14	1.82E−06
S_front_sup	2.47±0.13	2.55±0.09	2.07E−07
S_intrapariet&P_trans	2.07±0.10	2.14±0.11	2.95E−05
S_oc_middle&Lunatu	1.87±0.13	1.94±0.15	2.56E−04
S_oc-temp_lat	2.35±0.18	2.45±0.19	1.93E−04
S_oc-temp_med&Lingual	2.18±0.16	2.26±0.13	1.43E−04
S_orbital_med-olfact	2.21±0.16	2.33±0.14	2.40E−06
S_orbital-H_Shaped	2.55±0.17	2.66±0.17	1.03E−05
S_precentral-inf-part	2.38±0.13	2.47±0.12	3.04E−06
S_precentral-sup-part	2.45±0.12	2.52±0.12	2.87E−05
S_suborbital	2.50±0.28	2.66±0.29	1.23E−04
S_temporal_inf	2.35±0.15	2.43±0.12	2.50E−04
S_temporal_sup	2.28±0.11	2.37±0.09	1.21E−08
Right			
G&S_occipital_inf	2.32±0.19	2.42±0.19	3.25E−04
G&S_subcentral	2.52±0.15	2.60±0.16	3.24E−04
G&S_transv_frontopol	2.59±0.23	2.70±0.20	2.25E−04
G&S_cingul-Ant	2.73±0.17	2.81±0.13	2.91E−04
G&S_cingul-Mid-Ant	2.72±0.16	2.80±0.12	2.51E−04
G&S_cingul-Mid-Post	2.59±0.14	2.68±0.13	1.37E−05
G_cingul-Post-dorsal	2.81±0.18	2.90±0.15	1.42E−04
G_front_inf-Opercular	2.62±0.15	2.72±0.16	4.00E−05
G_front_sup	2.93±0.16	3.05±0.14	6.89E−07
G_oc-temp_lat-fusifor	2.62±0.16	2.74±0.17	3.74E−07
G_oc-temp_med-Lingual	2.09±0.14	2.17±0.17	2.89E−04
G_precuneus	2.42±0.13	2.52±0.13	2.93E−07
G_temporal_middle	2.72±0.15	2.81±0.15	7.93E−05
Lat_Fis-post	2.31±0.12	2.38±0.12	7.94E−06
S_cingul-Marginalis	2.23±0.13	2.30±0.12	1.48E−04
S_circular_insula_sup	2.44±0.12	2.56±0.13	1.20E−10
S_front_inf	2.16±0.13	2.24±0.12	8.19E−06
S_front_middle	2.23±0.14	2.35±0.13	7.27E−09
S_front_sup	2.48±0.12	2.59±0.10	1.13E−10
S_intrapariet&P_trans	2.07±0.09	2.14±0.10	1.01E−06
S_oc-temp_med&Lingual	2.15±0.15	2.24±0.12	3.82E−06
S_orbital_lateral	2.11±0.20	2.24±0.19	2.54E−06
S_orbital_med-olfact	2.19±0.13	2.28±0.14	4.87E−06
S_orbital-H_Shaped	2.48±0.15	2.56±0.14	1.31E−04
S_postcentral	2.07±0.10	2.14±0.10	8.04E−06
S_precentral-inf-part	2.35±0.12	2.44±0.12	1.77E−06
S_precentral-sup-part	2.47±0.12	2.55±0.12	2.94E−05
S_subparietal	2.26±0.17	2.36±0.14	1.80E−05
S_temporal_inf	2.13±0.15	2.23±0.13	2.75E−06
S_temporal_sup	2.23±0.12	2.31±0.11	8.07E−06

### Correlation analysis of abnormal cerebral cortex thickness with clinical features

Spearman correlation analysis found that cortical thickness at multiple locations was associated with different clinical characteristics in non-NPSLE patients. The results are shown in Table 5.

**Table 5:** brain regions with mean cortical thickness correlated with clinical features (*P* < 0.05)

Indicators	Left hemisphere	The correlation coefficient	*P*-value	Right hemisphere	The correlation coefficient	*P*-value
course of disease (months)	S_precentral-inf-part	−0.207	0.032	S_precentral-sup-part	−0.215	0.025
				S_subparietal	−0.202	0.036
Urine protein(g/24h)	S_circular_insula_sup	−0.206	0.032	G&S_subcentral	−0.219	0.023
	S_front_sup	−0.193	0.045			
	S_intrapariet&P_trans	−0.197	0.041			
	S_precentral-sup-part	−0.249	0.009			
Glucocorticoid accumulation(*n*=84)	S_oc-temp_med&Lingual	0.222	0.042	-	-	-
CTX accumulation(*n*=25)	-	-	-	S_temporal_inf	0.513	0.005
HCQ accumulation(*n*=46)	S_oc-temp_med&Lingual	0.309	0.036	S_oc-temp_med&Lingual	0.304	0.040
ANA	G&S_frontomargin	−0.201	0.037	G_front_sup	−0.229	0.017
	G_pariet_inf-Supramar	−0.270	0.005			
ACL	-	-	-	-	-	-
LAC (*n*=81)	-	-	-	S_orbital_lateral	−0.226	0.042

### Correlation analysis of abnormal cerebral cortex thickness with disease activity and psychometric scale

Non-NPSLE patients were divided into subgroups according to predefined scoring criteria which analyzed the scores obtained on SLEDAI, HAMD, and HAMA assessment scales. Spearman correlation analysis found correlations between these assessment tools, except SLEDAI, and abnormal cortical thickness in various brain regions. The results are displayed in Table 6.

**Table 6:** brain regions with correlation between mean cortical thickness and scales (*P* < 0.05)

Scales	Brain areas	The correlation coefficient	*P-*value
SLEDAI-2k	-	-	-
MMSE	Right G&S_cingul-Ant	0.297	0.002
HAMA	Left G_occipital_middle	0.215	0.025
HAMD	Right G&S_cingul-Mid-Ant	0.190	0.049
	Right G_oc-temp_lat-fusifor	0.190	0.049
	Right G_oc-temp_med-Lingual	0.200	0.038
	Right S_cingul-Marginalis	0.214	0.026
	Right S_orbital_med-olfact	0.250	0.009
	Right S_subparietal	0.210	0.037

### Correlation between abnormal cerebral cortex thickness and anti-nuclear antibody spectrum

The point biserial correlation is used to analyze the correlation between autoantibodies (negative or positive) and the average cortical thickness of the abnormal brain regions. The results are displayed in Table 7.

**Table 7:** Correlation between mean cortical thickness and antibodies to extractable nuclear antigens (*P* < 0.05)

ENA	Left hemisphere	The correlation coefficient	*P*-value	Right hemisphere	The correlation coefficient	*P*-value
Anti-dsDNA antibodies	-	-	-	-	-	-
Anti-Sm antibodies	-	-	-	-	-	-
Anti-U1RNP antibodies	-	-	-	-	-	-
Anti-SSA52KD antibodies	G_front_inf-Triangul	0.222	0.021	S_circular_insula_sup	0.191	0.047
Anti-SSA60KD antibodies	-	-	-	-	-	-
Anti-SSB antibodies	-	-	-	-	-	-
Anti-Histones antibodies	-	-	-	-	-	-
Anti-P0 antibodies	-	-	-	S_temporal_inf	0.235	0.014
Anti-Nucleosome antibodies	S_oc_middle&Lunatus	−0.191	0.048	-	-	-
Anti-DNP antibodies	-	-	-	-	-	-

### Structural covariance networks analysis

**Fig. 3:**
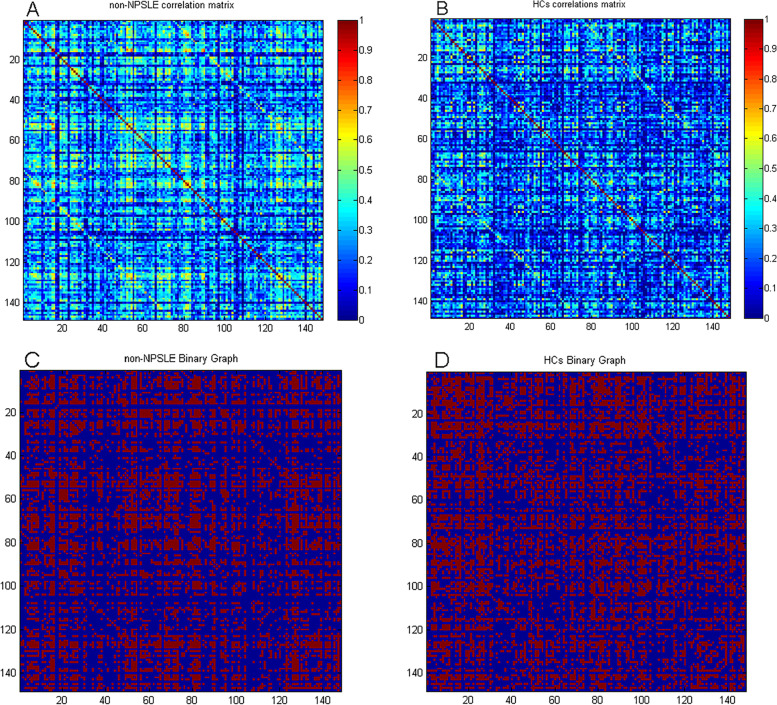
Correlation and binary matrices for non-NPSLE and healthy controls (HCs). Correlation matrices for non-NPSLE (**A**) and HCs (**B**), and binary adjacency matrices thresholded at Dmin (0.38) for non-NPSLE (**C**) and HCs (**D**). The color bar denotes the correlation coefficient and represents the strength of the connections

A correlation matrix and binary matrix for non-NPSLEs and healthy controls (HCs) can be seen in Fig. 3.

### Between-group differences in global network measures

**Fig. 4:**
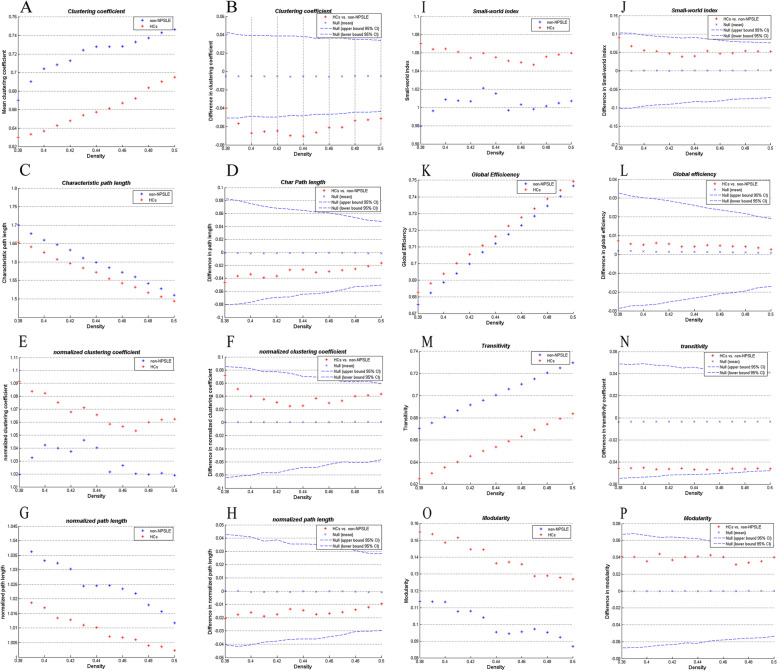
Global network measures of two groups, and between-group differences in these measures. Clustering coefficient (**A, B**), characteristic path length (**C, D**), Gamma (**E, F**), Lambda (**G, H**), small-world index (**I, J**), Global efficiency (**K, L**), transitivity (**M, N**), and modularity (**O, P**) of the non-NPSLE and HCs networks. The red * lying outside of the confidence intervals indicates the difference between the two groups in this density is significant (**B, D, F, H, G, L, N, P**). Except for a few densities, the clustering coefficient in HCs was found to be lower than that in non-NPSLE group (*P* < 0.05, FDR-corrected; **B**), the rest of the measures were not significantly different between the two groups. The non-NPSLE group does not follow the small-world attribute in densities of 0.38, 0.45, and 0.47

The correlation matrix illustrates a correlation in the cortical thickness of some brain regions in the two groups. (Fig. 1 A, B). We calculated and compared a series of global network measures at a range of densities (0.38–0.5 with an interval of 0.01) for the two groups, including clustering coefficient, characteristic path length, normalized clustering coefficient, normalized path length, small-world index, global efficiency, transitivity, and modularity and displayed them in Fig. 4. Except for Dmin, the clustering coefficient of the HC group in other densities was found to be lower than that of the non-NPSLE group (*P* < 0.05, FDR-corrected; Fig. 4 B); there were no significant differences between the groups on the rest of the measures (Fig. 4). It is worth noting that, unlike HC group, the non-NPSLE group does not follow the small-world attribute in a few densities (Fig. 4 I). Additionally, we also performed AUC analysis of network measures curves for the two groups. The results were consistent with the cross-density analysis, the clustering coefficient of HCs was significantly lower than that of the non-NPSLE group (*P* = 0.006).

### Between-group differences in regional network measures

**Fig. 5:**
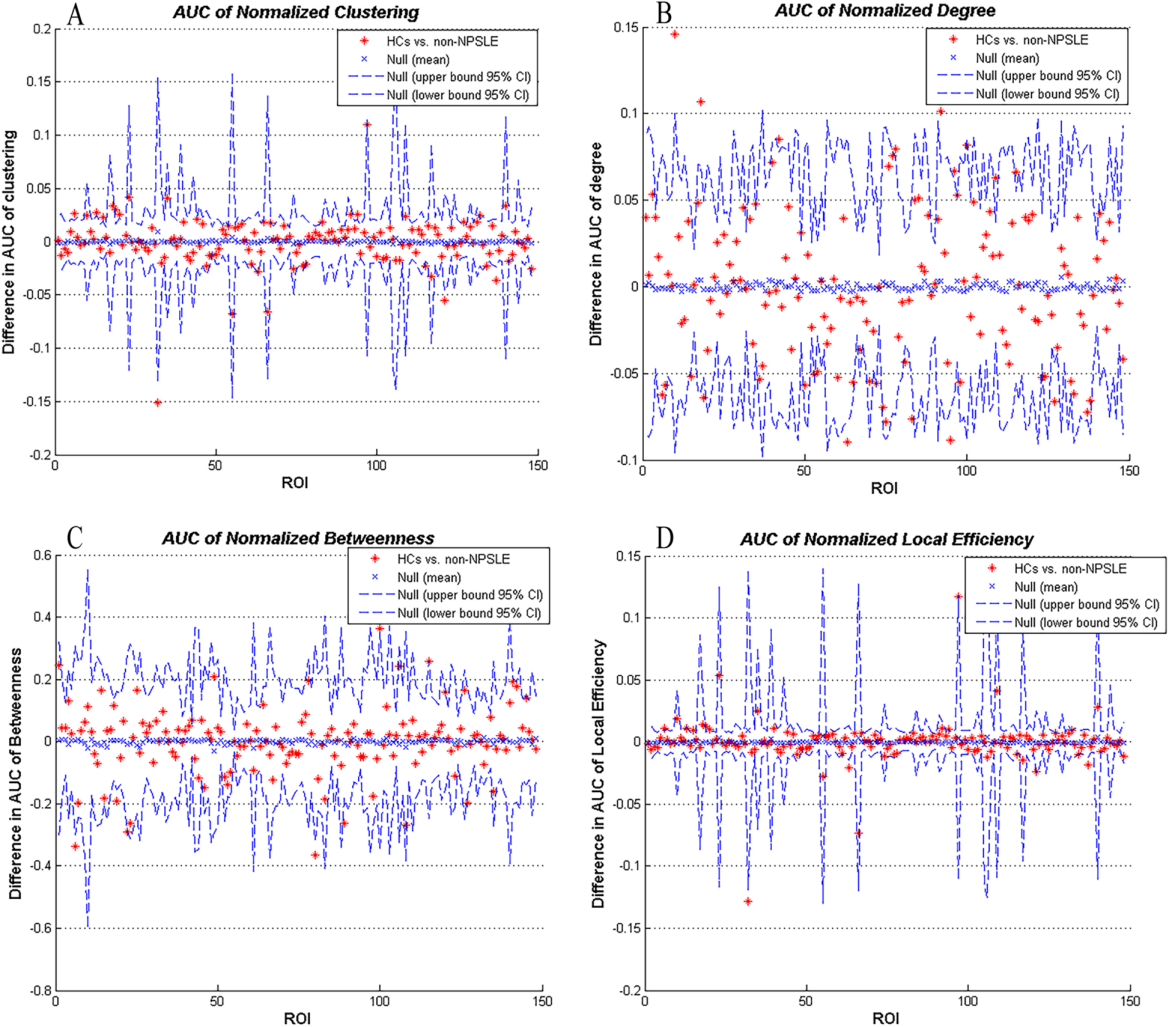
Between-group differences in normalized regional network (density range of 0.38–0.5 with an interval of 0.01) measuresfor each ROI: normalized clustering coefficient (**A**), degree (**B**), betweenness (**C**), and local efficiency (**D**). The red * lying outside of the confidence intervals indicate regions which the difference between the two groups in this density is significant. All regions survive following FDR correction (*P* < 0.05). non-NPSLE, non-NPSLE group; HCs, healthy controls. AUC, area under the curve

We performed AUC (density range of 0.38–0.5 with an interval of 0.01) analysis on four regional network measures, including clustering coefficient, degree, betweenness, and local efficiency. The results showed that part of the brain regions in the non-NPSLE group exhibit significant increase or decrease in the above measures compared with the HC group. See Table 8 and Fig. 5 for details.

**Table 8:** Between-group differences in regional network measures

	Non-NPSLE < HCs	Non-NPSLE > HCs	*P*-value
Clustering coefficient	lh_G_occipital_middle rh_S_front_middle lh_S_front_sup lh_G&S_cingul-Ant rh_S_oc_sup&transversal	rh_S_postcentral rh_S_orbital_med-olfact G_subcallosal rh_S_oc-temp_lat lh_S_circular_insula_sup rh_G&S_subcentral rh_Lat_Fis-post rh_S_circular_insula_ant lh_S_oc-temp_lat lh_S_orbital_med-olfact	0.05
Degree	lh_G_insular_short lh_G_cingul-Post-ventral rh_G_insular_short rh_G_temp_sup-Plan_polar rh_Lat_Fis-post lh_Pole_occipital lh_S_circular_insula_sup rh_G_oc-temp_med-Lingual rh_G&S_subcentral lh_G_temp_sup-Plan_polar rh_G_oc-temp_med-Parahip	lh_S_front_sup rh_G_oc-temp_lat-fusifor rh_S_front_middle rh_G_cingul-Post-dorsal rh_G&S_cingul-Mid-Ant lh_G_occipital_middle rh_S_circular_insula_sup lh_S_orbital_med-olfact lh_G_front_middle	0.05
Betweenness	lh_Pole_occipital rh_G_pariet_inf-Supramar	rh_G_front_middle lh_G_occipital_middle rh_G&S_cingul-Ant rh_S_front_middle lh_G&S_cingul-Ant	0.05
Local efficiency	lh_G_occipital_middle lh_G&S_cingul-Ant rh_S_oc_sup&transversal rh_G_occipital_sup	rh_S_orbital_med-olfact lh_S_orbital_med-olfact lh_G_subcallosal rh_Lat_Fis-post	0.05

*non-NPSLE*, non-NPSLE group; *HCs*, healthy controls; *lh*, left hemisphere; *rh*, right hemisphere. All regions survive following FDR correction (*P* < 0.05)

### Network hubs

The results show that non-NPSLE groups and HC groups significantly differ in the number of hubs as well as hub distribution. Specifically, there are a total of 7 hubs in the non-NPSLE group, respectively left G&S_cingul-Ant, left G_oc-temp_med-Lingual, left S_front_inf, right G&S_cingul-An, right G_front_middle, right G_temp_sup-Lateral, and right S_oc-temp_med&Lingual. The HCs has 5 hubs, including left G_front_sup, left S_circular_insula_sup, right G_pariet_inf-Supramar, right Lat_Fis-post and right S_front_inf.

## Discussion

This is the first cortical thickness study for non-NPSLE patients that uses both the SBM and ROI analysis. We also performed correlation analyses on the cortical thickness abnormalities of different brain regions obtained by the ROI method and a large number of clinical indicators. Finally, we constructed and analyzed the SCNs of cortical thickness in non-NPSLE patients for the first time, and discovered structural network abnormalities in non-NPSLE patients. Specifically, we found that non-NPSLE patients exhibit the following: (1) abnormal cortical thickness: the results of the two analytical methods both show that non-NPSLE patients have a wide range of brain regions with cortical thinning when compared with HCs; (2) multiple clinical indicators, disease activity, and mental scale results are in correlation with these abnormal brain regions; (3) changes in global network measures include the improvement of clustering coefficient, the breakdown of small-world attributes, fluctuating regional network measures in some of the brain regions, and changes in both the number and distribution of different network hubs. The above findings may provide meaningful information to deepen our understanding of SLE brain damage and explain the underlying mechanism of non-NPSLE-associated early neuropsychiatric abnormalities.

Cortical thickness is one of the most important indicators of brain structure analysis. In this study, before the appearance of obvious neuropsychiatric symptoms and conventional MRI abnormalities in non-NPSLE patients, extensive cortical thinning of the bilateral cerebral hemispheres was observed, which suggests that the cerebral cortex of non-NPSLE patients have undergone significant subclinical changes before evolving into NPSLE. Zivadinov et al. [43] conducted multimodal neuroimaging studies on lupus patients and found that cortical atrophy was the most relevant measurement index for central nervous system involvement in SLE. Similar SBM studies for SLE also found that SLE patients have thinner cortical thickness in multiple brain regions [9, 19, 20, 44]. Many previous studies [10, 45–47] have also verified the cerebral cortical atrophy in SLE patients by VBM and other different technical modalities; therefore, cortical thickness can be regarded as one of the imaging biomarkers for structural changes in SLE patients.

Although cortical atrophy in SLE patients has been extensively reported, the mechanisms of brain injury related to SLE are complex and the exact mechanisms have not been elucidated [48, 49]. Present understanding in the matter is that multiple interrelated mechanisms attributed to underlie SLE-related brain damage include blood-brain barrier dysfunction, vascular inflammation, thrombosis, vascular occlusion caused by atherosclerotic changes, neuroendocrine imbalance, tissue and neural damage mediated by autoantibodies (such as anti-ribosome P0 antibody, ACL), and proinflammatory factors (such as IL-1, IL-6, IL, 8, and TNF-α) combine to produce neuronal loss [50–53]. And progressive diffuse neuronal loss will eventually lead to the atrophy of and, consequently, thinning of cerebral cortex observed by structural magnetic resonance in this study.

In this study, the SMB and ROI methods were applied on the same group of subjects to compare cortical thickness based on the DS atlas. SBM analysis with post-correction (Monte-Carlo simulation cluster analysis with 1000 permutations, cluster-wise threshold of *P* < 0.05, and cluster-forming threshold of *P* < 0.001), revealed persistent significant cortical thinning in 34 clusters in non-NPSLE, which reflects the severity and diffuse nature of non-NPSLE-related brain damage. Prior SBM-based cerebral cortex studies in lupus patients have used FreeSurfer default DK atlas without exception. There has been no previous study separately on the non-NPSLE subgroup of SLE. Some brain regions with thin cortex identified here are well reported, such as the left supramarginal gyrus of NPSLE is thinner than control groups [20], the left superior temporal gyrus of SLE patients with episodic memory deficit is thinner than control groups [19], and the left superior parietal cortex of NPSLE is thinner than non-NPSLE [9], but newer abnormal brain areas have been discovered for the first time. We consider that this perceived discrepancy can be explained by differences in study population and the use of brain anatomy atlas. Current and prior studies suggest that the patterns of cortical involvement may be unique to subgroups of NPSLE; however, specific differences remain to be verified. In ROI analysis, we found more abnormal brain regions (57 regions in bilateral hemispheres), all of them showed cortical thinning in the non-NPSLE group, and 19 brain regions overlapped with SBM results. Incomplete congruency between the two results reflects that the anatomical regions defined according to the structural boundary may not correspond to the pathophysiological indicators of SLE patients’ cortex. Similar results have also been found in currently depressed study [54]. SBM is perhaps the more suitable, of the two methods, for detecting cortical thickness alterations that do not fit perfectly into predefined regions, but the superiority of one method over the other in detecting cortical abnormalities in SLE can only be established by further verification.

In this study, course of the SLE was found to be negatively correlated with cortical thinning in some brain regions, and no positive correlation was found, which also reaffirms the results of previous studies related to SLE. Cerebral injury associated with SLE seems to be a continuous process, and analysis of cortical thickness can be used as a clinical indicator for the evaluation of brain injury in SLE with varying disease courses. Similar conclusions have been reported in studies of type 2 diabetes [55]. Quantitative analysis of urine protein is the main clinical indicator for detecting lupus nephritis. Although the mechanism is not fully understood, the currently accepted view is that immune-mediated inflammation is the main cause of lupus nephritis [56]. Cortical thickness of some brain regions is negatively correlated with the range of proteinuria, suggesting a certain degree of correlation between lupus nephritis and lupus brain damage. Correlation analysis also found that the cumulative dosage of several commonly used immunosuppressants is positively correlated with the average cortical thickness of multiple areas of the brain, suggesting that these immunosuppressants may have a potential protective effect on cortical thickness. Our previous study also found that compared with the patients who had never received immunosuppressive therapy, immunosuppressive therapy of SLE patients with average whole-brain white matter volume tends to increase [57] and that the immune inhibitors nerve protection mechanism may be the result of the reduced nerve injury due to vasculitis. The pros and cons of long-term immunosuppressive therapy need to be clarified by more prospective studies.

The presence of multiple autoantibodies in serum is one of the most prominent features of SLE, and some autoantibodies may also play a key role in SLE-associated central nervous system injury. Serum levels of brain reactive antibodies in SLE patients are unrelated to neuropsychiatric symptoms, but their levels in cerebrospinal fluid are proven to be significantly related to neuropsychiatric symptoms. Therefore, the destruction of BBB is considered to be crucial for autoantibodies to enter the central nervous system. At present, the well-studied brain reaction autoantibodies associated with SLE include anti-N-methyl-d-aspartate receptors (NMDAR), anti-ribosome P0, microtubule associated protein 2 (MAP-2), matrix metalloproteinase 9 (MMP-9), RO anti body [58], and anti U1RNP and anti-phospholipid (APL) antibodies [59–61]. This study showed that serum ANA, LAC, and anti-nucleosome antibody were negatively correlated with cortical thickness in some brain regions, confirming their brain-damaging effect. However, serum anti-SSA52KD antibody and anti-P0 antibody were positively correlated with cortical activity in some brain regions, which was contrary to previous knowledge. At present, there are few studies on the exact relationship between anti-nuclear antibody spectrum and brain structural abnormalities. The specific mechanism by which autoantibodies exert effects on brain structure changes in SLE needs to be determined by future cerebrospinal fluid antibody research and animal experiments.

SLEDAI-2k has important value in the overall assessment of lupus disease activity and has reached a consensus among experts in the field of lupus research; it is widely used in clinical evaluation and scientific research of SLE. This study did not find a significant linear correlation between SLEDAI and cerebral cortex thickness, and none of the existing SLE cortical studies involved SLEDAI. In the diffusion-weighted magnetic resonance study, the SLEDAI-2k score also showed no correlation with mean diffusion or fractional anisotropy [62], while SLE brain studies have reported SLEDAI score and regional WM volume for the right internal capsule and left internal capsule [57]. On the one hand, these differences indicate that the disease activity in a certain period may indeed be irrelevant to the changes in brain structure caused by the long-term disease process. On the other hand, this again reflects the ambiguity of the current mainstream SLE disease activity assessment system for neuropsychiatric assessment and its incompatibility with the current rapidly changing advanced neuroimaging technology. The importance of magnetic resonance in the diagnosis and evaluation of neuropsychiatric lupus has also been valued by rheumatologists [63].

Previous studies have observed decreased white and grey matter in SLE patients with cognitive dysfunction in comparison to those with moderate cognitive impairment [45]. Our previous research also found that MMSE score is positively correlated with gray matter volume [10]. In this study, 63% of the subjects had normal cognitive function, whereas 32% had mild and 5% had moderate cognitive impairment; no subject has severe cognitive impairment. There is a positive correlation between MMSE grade and the cortical thickness of right G&S_cingul-Ant. The cingulate gyrus is part of the limbic system of the brain, and its functions involve emotion, learning, and memory. Clinical studies have shown cingulate abnormalities in many cases, including schizophrenia, depression, post-traumatic stress disorder, mild cognitive impairment, and Alzheimer’s disease. Cognition is the functional result of learning and memory process produced by the interaction of various neurotransmitters, transcription factors, cytokines, and chemokines between neurons, astrocytes, glial cells, and immune cells. Cognitive dysfunction is a common phenomenon in SLE [64, 65]; however, its pathological mechanism still needs additional study. Some studies have found that there are multiple factors related to anxiety and depression in SLE, including certain specific autoantibodies, nerve damage, the presence of rash, the concentration of certain cytokines, pain and disability caused by pain, and socioeconomic status [66–68]. These different findings suggest that depression and anxiety in SLE patients may be mediated by a complex mix of biosocial and environmental factors. This study found that some non-NPSLE patients have anxiety and/or depression, as shown in Fig. 1, and HAMA and HAMD grades were positively correlated with cortical thickness in some brain regions, respectively. In a MRI resonance study of primary anxiety and depression, there was no significant correlation between anxiety symptoms and brain structural indicators, while depression symptoms were related to the thinner cortical thickness of some brain regions [69]. A recently published large-scale analysis combining global data shows that the brain structure of patients with generalized anxiety disorder has not changed significantly [58]. At present, in SLE, the relationship between anxiety, depression, and cerebral cortex thickness is still unclear, and more targeted research is needed to verify it in the future.

Recently, brain connectivity has been increasingly used to study the pathological mechanisms of brain involvement. For SLE, studies have also found abnormalities in functional connectivity [42] and white matter connectivity [70, 71]. SCN studies on SLE cortex are yet unavailable. The first part of this study also shows that changes in cortical thickness in a non-NPSLE brain are widespread and not confined to a few brain regions. Furthermore, we investigated SCNs based on cortical thickness in the non-NPSLE context in order to explore the multivariable network relationship between different neuroanatomical regions.

Between-group comparisons of global network measures primarily found that except for Dmin, the clustering coefficient of the HCs in other densities was lower than that of the non-NPSLE patients (Fig. 4 B), while other measures were not significantly different (Fig. 4). Clustering coefficients are defined as the ratio of edges between nodes in a neighborhood divided by the number of edges between them; it reflects the degree of interconnection interconnectivity between network nodes and their neighbors. The changes in clustering coefficient of SCNs in different diseases are heterogeneous; for example, it is increased in tinnitus [72] and decreased in type 2 diabetes mellitus [73], whereas no significant change is seen in vertically infected HIV adolescents [74]. The extensive reduction of cortical thickness and the enhancement of the interaction between abnormal cortex may be characteristics of non-NPSLE brain damage. Presence of initial brain lesions and their progression needs to be explored in future studies. The small-world feature represents two fundamental features of a brain’s information-processing system: functional separation and functional integration; the former refers to the ability of closely connected brain regions to process information, and the latter refers to the ability of arbitrarily distributed brain regions to transmit information. In this network structure, local adjacent brain regions are closely connected, and a small number of connections are created between any two brain regions for rapid communication. By achieving the balance between local information processing and whole-brain transmission, not only will functional classification and integration be efficient but it also reduces the cost of maintaining efficient communication [75, 76]. In this study, the non-NPSLE group does not follow the small-world attribute in a few densities (small-world index<1, see Fig. 3 I), reflecting the sub-optimization of non-NPSLE SCNs.

For comparison of regional network measures between groups, we performed AUC (density range of 0.38–0.5 with an interval of 0.01) analysis on normalized clustering coefficient, degree, betweenness, and local efficiency of each brain region in the two groups (see Fig. 5). We found that some brain regions displayed increased or decreased values for the above given measures, reflecting the extensive and obvious changes in the SCN attributes of the non-NPSLE cortex in the local brain regions. DS atlas, utilized to construct the network in this study, divides the cerebrum more finely and into more regions than DK atlas adopted by many other similar studies done on other diseases [39, 77, 78]. This explains why the network measures found in this study have changed in more nodes. On the other hand, perhaps like the reduction in cortical thickness, the abnormalities of regional SCNs in large areas of the brain and the compensatory changes in corresponding brain areas are the characteristics of non-NPSLE. This will also require SLE magnetic resonance data from other research centers in the future to assist in verification.

The hubs of non-NPSLE and HCs are different both in number and location.

Of the 7 hubs of non-NPSLE, 3 are located in the frontal lobe, 2 are in the anterior cingulate cortex, and 2 are in the temporal lobe. Of the 5 hubs of HCs, 2 are in the supramarginal gyrus, 2 are in the temporal lobe, and 1 is in the insula. The main area for hubs increases, and studies have found that the anterior cingulate cortex and temporal lobe are related to emotions and learning [79–81]. However, the pathological mechanism of the changes in these locations has not yet been elucidated in SLE. The increase of hubs in these regions may be a compensatory supplementation. No common hubs were found between non-NPSLE and HCs. The increased number of hubs associated with changes in the location may be a feature of the non-NPSLE cortical thickness covariant network. These findings may also be caused by individual differences between subjects.

This study also has some limitations. First of all, the field strength of the magnetic resonance scanner used in this study (1.5T) is lower than that currently mainstream in brain imaging research (3.0T), so our results may be biased due to the smaller signal-to-noise ratio. We actually started this research 10 years ago and continued the same 1.5-T MRI scanner and scanning parameters to build the database. In this study, we conducted strict quality control, including manual visual inspection and software quality control. We added detailed quality control information in the supplementary materials. We also hope to use new magnetic resonance scanners in the future to obtain more accurate results. Secondly, cross-sectional design of this study makes it impossible for us to describe the dynamic changes in non-NPSLE cerebral cortex and SCNs along with disease progression; this can only be achieved with longitudinal research. Thirdly, when performing SCN analysis, each group has only one network, instead of each subject having a separate network, so we cannot test the relationship between network parameters and clinical measurements.

## Conclusion

In conclusion, extensive and obvious reduction in cortical thickness and abnormal topological organization of SCNs are observed in non-NPSLE patients. The observed abnormalities may not only be the realization of brain damage caused by the disease, but also the contribution of the compensatory changes within the nervous system.

## Data Availability

The datasets generated and analyzed during the current study are not publicly available due to protection of individuals’ privacy but are available from the corresponding author on reasonable request.

## Abbreviations

non-NPSLE: Non-neuropsychiatric systemic lupus erythematosus
SCNs: Structural covariance networks
HCs: Healthy controls
SBM: Surface-based morphometry
ROIs: Regions of interest
NPSLE: Neuropsychiatric lupus erythematosus
SLE: Systemic lupus erythematosus
MRI: Magnetic resonance imaging
CT: Cortical thickness
DK: Desikan–Killiany
DS: Destrieux
ACR: American College of Rheumatology
GC: Glucocorticoids
CTX: Cyclophosphamide
HCQ: Hydroxychloroquine
ANA: Autoantibodies such as anti-nuclear antibody
ASmA: Anti-Smith antibody
Anti-dsDNA: Anti-Double-stranded DNA
ARPA: Anti-ribosomal P0 antibody
LAC: Lupus anticoagulant material
ACL: Anti-cardiolipin antibody
IIFA: Indirect immunofluorescence
SLEDAI-2K: Systemic Lupus Erythematosus Disease Activity Index 2000
MMSE: Mini-Mental State Examination
HAMD: Hamilton Depression Scale
HAMA: Hamilton Anxiety Scale
TE: Echo time
TR: Repetition time
FWHM: Full-width half maximum
GLM: General linear model
VBM: Voxel-based morphometry
NMDAR: Anti-N-methyl-d-aspartate receptors
MAP-2: Microtubule associated protein 2
MMP-9: Matrix metalloproteinase 9
APL: Anti-phospholipid
